# Comparing the similarity and spatial structure of neural representations: A pattern-component model

**DOI:** 10.1016/j.neuroimage.2011.01.044

**Published:** 2011-04-15

**Authors:** Jörn Diedrichsen, Gerard R. Ridgway, Karl J. Friston, Tobias Wiestler

**Affiliations:** a Institute of Cognitive Neuroscience, University College London, London, UK; b Wellcome Trust Centre for Neuroimaging, University College London, London, UK

## Abstract

In recent years there has been growing interest in multivariate analyses of neuroimaging data, which can be used to detect distributed patterns of activity that encode an experimental factor of interest. In this setting, it has become common practice to study the correlations between patterns to make inferences about the way a brain region represents stimuli or tasks (known as representational similarity analysis). Although it would be of great interest to compare these correlations from different regions, direct comparisons are currently not possible. This is because sample correlations are strongly influenced by voxel-selection, fMRI noise, and nonspecific activation patterns, all of which can differ widely between regions. Here, we present a multivariate modeling framework in which the measured patterns are decomposed into their constituent parts. The model is based on a standard linear mixed model, in which pattern components are considered to be randomly distributed over voxels. The model allows one to estimate the true correlations of the underlying neuronal pattern components, thereby enabling comparisons between different regions or individuals. The pattern estimates also allow us to make inferences about the spatial structure of different response components. Thus, the new model provides a theoretical and analytical framework to study the structure of distributed neural representations.

## Research Highlights

► Method to decompose multivariate voxel patterns into different components. ► Yields unbiased estimates of pattern correlations that can be compared across regions. ► Allows the study of the spatial structure of different pattern components.

## Introduction

Recent years have seen a rapid growth of multivariate approaches to the analysis of functional imaging data. In comparison to more traditional mass-univariate approaches (Friston et al., 1995; Worsley et al., 2002), multivariate pattern analysis (MVPA) can reveal changes in distributed patterns of neural activity (Haxby et al., 2001; Haynes and Rees, 2005a, b). A particularly interesting variant of these approaches can be described as “local” multivariate analyses (Friman et al., 2001; Kriegeskorte et al., 2006). Rather than using the whole brain (Friston et al., 1996), groups of neighbouring voxels (or cliques) are analyzed. Cliques can be selected using anatomically based regions-of-interest (ROI), or using a so-called search light, where a spherical ROI is moved across the brain to generate a map of local information content (Kriegeskorte et al., 2006; Oosterhof et al., 2010a). The key question addressed by these analyses is whether a group of voxels encodes a stimulus dimension or experimental factor. This involves demonstrating a significant mapping between the experimental factor and the distributed measured pattern (encoding models) or vice versa (decoding or classification models) (Friston, 2009). This can be done using cross-validation (Misaki et al., 2010; Norman et al., 2006; Pereira et al., 2009) or Bayesian approaches (Friston et al., 2008).

Multivariate analyses not only show that a variable is encoded in a region, but can also tell us how this variable is encoded. One common approach is the so-called representational-similarity analysis (Kriegeskorte et al., 2008), which investigates the correlations (or some other similarity metric) between mean patterns of activations evoked by different stimuli or task conditions. For example, one region may show very similar patterns for condition A and B and for C and D, but large differences between these pairs of conditions. This indicates the dimensions over which pattern activity is modulated by different experimental manipulations and therefore how the population of neurons may represent a factor of interest. Such an approach would be especially powerful if one could compare between-pattern correlations from different regions, thereby revealing regional differences in representation and (by inference) computational function.

However, the comparison of correlations (calculated between two conditions across voxels) across different regions is statistically invalid. This is because sample correlation coefficients are not a direct measure of the underlying similarity of two patterns, but are influenced by a number of other factors. For example, if the BOLD signal is noisier in one region than another (e.g. due to higher susceptibility to physiological artifacts, etc.) correlations will tend to be lower. Furthermore, the criteria by which one selects voxels over which to compute the correlation will strongly influence their size: If one picks a set of highly informative voxels the correlation between two patterns may be very high, but will decrease as more uninformative voxels are included. Finally, a particularly high correlation between two patterns does not necessarily indicate that the two specific conditions are encoded similarly; it could simply mean that there is a common (shared) response to any stimulus of this class. For these reasons, differences between sample correlations are largely un-interpretable. Thus, the best we can currently do is to compare the rank-ordering of correlations across different regions (Kriegeskorte et al., 2008), thereby disregarding valuable quantitative information.

Here, we present a generative model of multivariate responses that addresses these issues and furnishes correlations that are insensitive to the level of noise, common activation, and voxel-selection. The model assumes that the observed patterns are caused by a set of underlying pattern components that relate to the different experimental conditions or noise. Critically, and in contrast to traditional univariate approaches, these pattern components are not considered to be vectors of unknown constants (one for each voxel). Rather, they are conceptualized as a random variable with a probability distribution across voxels with a certain mean, variance (or power), and covariance (or similarity) with other patterns. The core idea of our approach is to estimate the variances and covariances of the underlying pattern components directly, using the sample covariance of the observed data. This allows us to derive unbiased estimates of the true correlation coefficients among the distributed condition-specific pattern components. The implicit random-effects model for distributed responses is inherently multivariate as it uses variance–covariance information over groups of voxels—in contrast to the univariate fixed-effects model, in which we would estimate the pattern associated with a condition by calculating the mean response to that condition and subtract the mean pattern across conditions. Our model accommodates the fact that part of this average response is caused by noise and adjusts its estimates of variances, covariances and correlations accordingly.

As in a Gaussian process model (Rasmussen and Williams, 2006), we recast the problem of estimating response patterns into the problem of estimating the variance–covariance of the underlying components. Because we parameterize the model in terms of variances and covariances, the correlation between different patterns, induced under different experimental conditions, is estimated in an explicit and unbiased fashion and can be used as a summary statistic for subsequent hypothesis testing about representational similarities. Furthermore, the approach can handle a large number of voxels with no increase in computational overhead or identifiability problems. This is because we focus on the second-order behaviour of the data (power or variance) as opposed to the first-order behaviour (patterns or mean).

This paper comprises four sections. The first presents our pattern component model and shows how covariances among patterns can be specified and estimated. In the next section, we use a simple one-factorial design with 3 different stimuli to show how our method robustly accommodates different levels of noise or common activations, to furnish unbiased (corrected) correlation coefficients that can then be used for further analysis. Thirdly, we provide a more complex example that uses a two-factorial 4 × 2 design, and show how our method can be used to test specific hypothesis about how main effects and interactions are expressed in terms of distributed patterns. Using this experimental design, we then provide an illustrative application to real data. We also show that spatial correlations between voxels do not bias our covariance component estimation process. Finally we show that we can recover information about the spatial smoothness for each of the underlying pattern components, thereby characterising not only the similarity, but also the spatial structure of the underlying neural representations. Appendix A provides a detailed presentation of the estimation algorithm and methods for accelerating its computations.

## The pattern component model

### Model structure

**Fig. 1:**
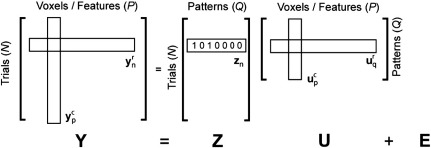
Covariance component model. The data (*Y*) comprise the activations over *P* voxels and *N* trials. The observed patterns (**y**_*n*_^*r*^) are generated from a set of *Q* unknown or hidden pattern components **u**_*q*_^*r*^ and noise *E*. The hidden patterns **u**_*q*_^*r*^ are modelled as random effects over the voxels, such that the columns **u**_*p*_^*c*^ are distributed normally with variance–covariance matrix **G**. This matrix encodes the similarity structure of the different patterns.

Let **Y** = [**y**_1_^*r*^,..., **y**_*N*_^*r*^]^*T*^ ∈ *ℜ*^*N* × *P*^ be the data for N trials, each of which contains *P* voxels or features (Fig. 1). We will assume here that the data are summary statistics (e.g., regression coefficients) from a first-level time-series analysis, for example, the activation for each behavioural trial in an event-related fMRI paradigm^1^. In other words, we assume that each row (**y**^*r*^) of our data matrix **Y** is the measured pattern over spatial features (e.g., voxels), and that the rows constitute independent samples from different trials. For simplicity, we will assume that effects of no interest have been removed from the summary data. We can also split the data into *P* column vectors, each encoding the activity of a particular voxel for the *N* trials: **Y** = [**y**_1_^*c*^,..., **y**_*P*_^*c*^]. For convenience of notation, both **y**^*c*^ and **y**^*r*^ are column vectors.

Each trial has an associated experimental or explanatory variable **z**_*n*_ ∈ *ℜ*^*Q* × 1^. This vector may consist of indicator variables denoting the experimental condition in a one- or multi-factorial design. Alternatively, **z**_*n*_ may contain a set of parametric variables. We assemble the experimental variables into a design matrix, with each row of the matrix corresponding to a single trial and each column to an experimental effect, **Z** = [**z**_1_,..., **z**_*N*_]^*T*^ ∈ *ℜ*^*N* × *Q*^.

We start with a model that assumes the data are generated as a linear combination of *Q* pattern components plus some noise (see Fig. 1).

(1) Y=ZU+E

The rows of the matrix **U** = [**u**_1_^*r*^,..., **u**_*Q*_^*r*^]^*T*^ : = [**u**_1_^*c*^,..., **u**_*P*_^*c*^] ∈ *ℜ*^*Q* × *P*^ are the underlying pattern components associated with the *Q* experimental effects. **E** ∈ *ℜ*^*N* × *P*^ is a noise matrix, in which terms for single voxels (the columns of **E**) are assumed to be independent and identically distributed (i.i.d.) over trials, i.e. $\varepsilon_{p}^{c}\sim N(0,I\sigma_{\varepsilon }^{2})$. Note that we have not made any assumption about the dependence or independence of these effects in the spatial domain (see Covariance between voxels).

We have now *Q* unknown pattern components and *N* unknown noise components that we wish to estimate. Direct estimation is impossible, because we have only *N* observed patterns as data. The novel approach we adopt is to consider the pattern components to be randomly distributed across voxels and to estimate not the pattern components directly but the energy and similarity (variances and covariances) associated with those patterns. Given these variances and covariances, we can then obtain the random-effects estimates of the patterns.

Thus, we assume that across the *P* voxels, the columns of **U** are distributed normally with mean **a** and variance–covariance matrix **G**.

(2) $$u_{p}^{c}\sim N\left(a,G\right)$$

Because each pattern component has its own mean value, the estimates of **a** do not depend on **G**. Thus we can estimate **a** using the pseudo-inverse of **Z** as $a=Z^{+}\sum_{p}y_{p}^{c}/P$ and simply subtract Za from each $y_{p}^{c}$. Without a loss of generality, we can therefore assume that the mean-subtracted column vectors $y_{p}^{c}$ have a normal distribution with mean **0** and variance–covariance:

(3) $$\text{var}\left(y_{p}^{c}\right)=\text{var}\left(Zu_{p}^{c}+\varepsilon_{p}^{c}\right)=Z\text{var}\left(u_{p}^{c}\right)Z^{T}+\text{var}\left(\varepsilon_{p}^{c}\right)=ZGZ^{T}+I\sigma_{\varepsilon }^{2}$$

This is a random-effects model, in which we have converted the problem of estimating the unknown pattern components into the problem of estimating the unknown variance–covariance matrix $G\in {ℜ}^{Q\times Q}$ that underlies the expression of the Q pattern components. The leading diagonal terms of **G** parameterize the overall energy or variance over voxels associated with each component, while the off-diagonal terms encode the similarity among components. Once we have obtained an estimate of **G**, the random pattern components can be estimated using the best-linear-unbiased predictor:

(4) $$U=GZ^{T}{\left(ZGZ^{T}+I\sigma_{\varepsilon }^{2}\right)}^{-1}Y$$

### Estimation of **G**

If **G** is unconstrained, our model is the standard random-effects model. In such cases, an Expectation–Maximization (Laird et al., 1987) or Newton–Raphson (Lindstrom and Bates, 1988) algorithm can be used to compute maximum-likelihood or restricted-maximum likelihood estimators for the variance parameters. As we will see in the following, many applications demand certain constraints on **G** that embody structural assumptions about the underlying pattern components. For example, one may want to constrain the variances for all levels of one factor to be the same, or one may want to impose the constraint that some components are uncorrelated (see examples below). Thus, ideally, we should be able to specify an arbitrary set of linear constraints on **G**.

In estimating the elements of the constrained **G**-matrix, we need to ensure that **G** is a true covariance matrix; i.e. it is positive definite. Here, we solve this problem by expressing **G** as $AA^{T}$, and by imposing the linear constraints on **A**, rather than on **G**. This is achieved by constructing **A** as a linear combination of basis matrices. The full EM-algorithm to estimate **A** is presented in the Appendix A.

## Example of a one-factorial design

### Effect of noise on similarity analysis

**Fig. 2:**
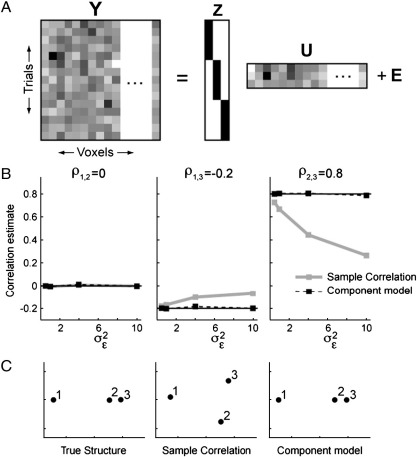
Example of a one-factorial design with 3 stimuli shows the influence of noise on sample correlations. (A) The five measures for each of the 3 conditions consist of the corresponding true pattern component U and noise E. (B) Dependence of sample correlations (gray line) and of the estimates from the component model (dashed line) on the noise variability (*σ*_*ε*_^2^). Correlations between stimulus 1 and 2, stimulus 1 and 3, and stimulus 2 and 3 are shown. The true value is indicated by a line. (C) Multi-dimensional scaling of similarity structure, based on 1-r as a distance metric. The true similarity structure can be represented as one dimension.

To give an illustrative example, let us consider a one-factorial experiment using 3 stimuli (Fig. 2A). The researcher may want to know, which of three pairs of stimuli are represented in a similar way; whether the similarity structure can be captured by one underlying dimension (that the region encodes), and how the similarity structure changes across regions.

In this one-factorial design with 3 levels, we can think of $u_{1}^{r}$,$u_{2}^{r}$, and $u_{3}^{r}$ as the three (unknown) pattern components encoding each level. Because the three conditions may be represented with different strengths, they may have different variances, with a high variance indicating a stronger response. Furthermore, each pair of pattern components may share a positive or negative covariance; i.e. they may be similar to each other or be partly inverse images of each other. These similarities correspond to the covariances *γ*_*i*, *j*_ between two pattern components. Thus in this simple case, our model would take the form:

(5) $$G≜\mathrm{var}\left[\begin{array}{c} u_{1}\\ u_{2}\\ u_{3} \end{array}\right]=\left[\begin{array}{ccc} \text{var}\left(u_{1}\right) & \text{cov}\left(u_{1},u_{2}\right) & \text{cov}\left(u_{1},u_{3}\right)\\ \text{cov}\left(u_{2},u_{1}\right) & \text{var}\left(u_{2}\right) & \text{cov}\left(u_{2},u_{3}\right)\\ \text{cov}\left(u_{3},u_{1}\right) & \text{cov}\left(u_{3},u_{2}\right) & \text{var}\left(u_{3}\right) \end{array}\right]=\left[\begin{array}{ccc} \sigma_{1}^{2} & \gamma_{1,2} & \gamma_{1,3}\\ \gamma_{2,1} & \sigma_{2}^{2} & \gamma_{2,3}\\ \gamma_{3,1} & \gamma_{3,2} & \sigma_{3}^{2} \end{array}\right]$$

The similarity of two pattern components is reflected in their true correlation:

(6) $$\rho_{i,j}=\frac{\gamma_{i,j}}{\sigma_{i}\sigma_{j}}$$

Because we do not have access to the true (population) correlations, a typical approach is to calculate the mean response patterns ${\bar{y}}_{i}^{r}$ for each stimulus and look at the sample correlations among them. Because each measurement is corrupted by noise, these sample correlations will be closer to zero than the true correlations. To illustrate this, we simulated an example in which the true patterns for stimuli 1 and 2 are represented independently of each other (*ρ*_1, 2_ = 0), patterns 1 and 3 are negatively correlated (*ρ*_1, 3_ = − 0.2), and patterns 2 and 3 are positively correlated (*ρ*_2, 3_ = 0.8). We simulated *n = 5* trials per condition for *P* = 100 voxels, setting the variance of the patterns to *σ*_*i*_^2^ = 1, and varying the noise variance *σ*_*ε*_^2^ between 0.5 and 10. As expected, the sample correlations become smaller (closer to zero) with increasing noise (Fig. 2B, gray line). Analytically, the expected value of the sample correlation between stimulus *i* and *j* under our model is:

(7) $$E\left(cor\left({\bar{y}}_{i}^{r},{\bar{y}}_{j}^{r}\right)\right)=\frac{\gamma_{i,j}}{\sigma_{i}\sigma_{j}+\sigma_{\varepsilon }^{2}/n}$$

Thus, while the rank ordering of the sample correlations is still interpretable, the absolute size of the correlation coefficients is not. This constitutes a problem if one tries to compare the correlations across different regions or individuals. Using the pattern component model (Fig. 2A, Eq. (5)), we are able to estimate the variance–covariance matrix of the hidden pattern components directly. By plugging these estimates into Eq. (6), we can then obtain corrected correlation coefficients. These provide the appropriate summary of relationships among the *Q* condition-specific pattern components, as opposed to the sample correlations, which are based on the fixed effects estimate (i.e. sample mean) for the underlying responses in each condition. The corrected estimates reflect the true size of the correlations for all stimulus pairs, independent of the level of noise (Fig. 2B, black dashed line). Correlation estimates from different regions can now be compared in a quantitative and meaningful way.

Furthermore, similarity structures can be analyzed and visualized using multi-dimensional scaling (Borg and Groenen, 2005). Here, one defines a distance metric between each pair of stimuli (here 1 − *ρ*), and attempts to find a space in which the stimuli can be arranged in such a way that their spatial distance best reflects this similarity. In our example, the true similarity structure can be visualized using a single dimension, with stimulus 2 and 3 being grouped together (see Fig. 2C, true structure). The similarity structure revealed by sample correlations, however, is very sensitive to the level of noise (Fig. 2C, sample correlations): with high noise, two dimensions are needed to represent the similarities, and the different stimuli appear to be equidistant from each other. Using our corrected estimates, the true one-dimensional structure is restored.

### Correcting for a common activation pattern

**Fig. 3:**
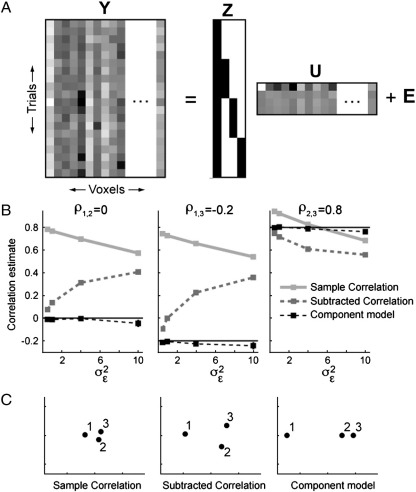
Example of one-factorial design of three conditions that share a common activation pattern. (A) The data consist of 5 measures of the control condition, which provides a measure of the common activation pattern (first row in matrix U), followed by 5 measurements for the 3 conditions each. (B) Due to the common activation, the sample correlations (light gray line) between mean patterns are much higher than the true correlations (line). Prior subtraction of the mean pattern of the control condition (dark gray dashed line) lowers the estimates, but still overestimates the correlation and underestimates the differences. Using the pattern component model (black dashed line), valid estimates can be obtained. (C) Multidimensional scaling based on the estimated correlation coefficients shows distortions of similarity structure for sample correlations between mean patterns.

In many cases, the measured activation pattern of different stimuli may be highly correlated with each other, because they share a common nonspecific factor. For example, in a visual experiment all stimuli may be preceded by a cue or may be followed by a response, both of which would elicit a distributed activation. We can think about this activation as a pattern component that has variance *σ*_c_^2^ over voxels and that is added to the measured activity pattern of each trial (Fig. 3A). When simulating data with *σ*_c_^2^ = 4 (all other simulation parameters as before), we indeed see that the sample correlations between all pairs of stimuli become highly positive (light gray line, Fig. 3B). When attempting to compare correlations from different individuals or regions, this is problematic, as different regions may show this common pattern in varying degrees.

To address this issue, one could introduce a control condition, which shares the nonspecific factors with all other conditions, but does not have any specific similarity with the conditions of interest. A typical approach would then be to subtract the mean activation pattern of the control condition from each of the condition-specific patterns and to calculate the sample correlation between these control-subtracted patterns. These correlations (dark gray dashed line, Fig. 3B) indeed correct for some of the positive correlation, bringing the correlation estimates closer to the true values. However, the measures are still biased. As the noise variance increases, the estimated correlations also increase. The reason for this behaviour is the following: The fixed-effects estimate of the common activation pattern (the mean pattern in the control condition) is itself corrupted by measurement noise. By subtracting the same random fluctuation from all the patterns, one introduces an artificial positive correlation between the ensuing residuals.

Thus, to correct for the common activation, a random-effects estimate of the common pattern is needed. In our pattern component model, we can conceptualize this common factor as a pattern component that is shared by all stimuli (first row of **U**, Fig. 3A), and that has variance *σ*_c_^2^. We assume here that this common component is uncorrelated with the pattern components that distinguish between different stimuli:

(8) $$G=\left[\begin{array}{cccc} \sigma_{c}^{2} & 0 & 0 & 0\\ 0 & \sigma_{1}^{2} & \gamma_{1,2} & \gamma_{1,3}\\ 0 & \gamma_{2,1} & \sigma_{2}^{2} & \gamma_{2,3}\\ 0 & \gamma_{3,1} & \gamma_{3,2} & \sigma_{3}^{2} \end{array}\right]$$

Thus, we define the stimulus-specific pattern components to be variations in activity that are orthogonal to the mean component, not stronger or weaker versions of the mean response. Our algorithm (see Appendix A) allows us to impose constraints on the variance–covariance matrix **G**, or more accurately, the square root (factor) **A** of this matrix. Thus, instead of explicitly estimating the common activation pattern and then subtracting it from the other patterns, we estimate the similarity structure of the stimuli directly, under the assumption that they share a common source of variance across voxels. The resulting estimates of the correlations correct for noise and the common activation pattern simultaneously (Fig. 3B). This correction makes it possible to compare the size of the correlations across different regions, even if these regions exhibit a common activation pattern to a different degree or have different levels of noise. Furthermore, the component model restores the true (one-dimensional) multidimensional similarity structure (Fig. 3C).

It may not always be possible to find a control condition that contains the nonspecific components and is equally dissimilar to all stimuli of interest. In such a case we can also introduce a common activation pattern into the model without measuring it separately in a control condition. This, however, generates an implicit ambiguity, as a positive correlation between the measured patterns could be explained either by a positive covariance between the stimulus-specific pattern components, or by a high variance of the common pattern component. To resolve this ambiguity, we then need to anchor the similarity scores by assuming that one or multiple pairs of pattern components associated with the stimuli of interest are uncorrelated, thereby introducing the necessary constraint into the **G** matrix. In the following 2-factorial example we will provide an example of such an approach (see also Eq. (A7)).

## Example using a 2-factorial design

### Accessing similarities across conditions

In this section, we further illustrate the use of our method for a more complex 2-factorial design, and show how the model can be used to test specific hypotheses about the structure of neural representations. In our example, a participant moved or received sensory stimulation to one of the four fingers of the right hand on each trial. Thus, Factor A was the experimental condition (movement vs. stimulation), while Factor B encoded which of the 4 fingers was involved. Overall, there were 8 experimental conditions (Wiestler et al., 2009), each repeated once in 7 imaging runs. In factorial designs like this, we can ask a number of questions: a) Does the region encode information about the finger in the movement and/or stimulation condition? b) Are the patterns evoked by movement of a given finger similar to the patterns evoked by stimulation of the same finger? c) Is this similarity greater in one region than another?

To answer question (a), we could use a standard multivariate test (e.g. CCA, Friston et al., 1996) or a classification and cross-validation procedure (Pereira et al., 2009); in which we train a classifier on the data from 6 runs, and then test whether the classifier can successfully “predict” from the activation patterns of the 7th run which finger was moved or stimulated. Similar approaches could also be used to answer question (b). Here we could train the classifier on patterns from the movement condition and then test the classifier on the stimulation condition (Oosterhof et al., 2010b). Alternatively, one can use representational-similarity analyses and test if there is a higher correlation between movement and stimulation patterns for the same finger, compared to different fingers. However, to answer question (c) this approach will not suffice: As we have seen, sample correlations are influenced by noise and strength of common activation, which makes direct comparisons across regions impossible.

To capture this more complex 2-factorial design in the pattern component model, let us first assume that all movement trials share a component (*u*_*α*[1]_), induced by the task. Similarly, there is an overall pattern component associated with sensory stimulation (*u*_*α*[2]_). These two components may also share a true covariance (*γ*_*α*_) that reflects common task activity (i.e. seeing the instruction cue). Thus, together these two pattern components encode the intercept and the main effect of condition (movement vs. stimulation). To capture the second factor of the experimental design, we assume that each finger has a specific pattern component associated with it, one for each experimental condition (*u*_*β*[*c*, 1, … 4]_ : *c* ∈ 1, 2). The variance of these components may be different for movement and stimulation conditions (*σ*_*β*[1]_^2^ vs. *σ*_*β*[2]_^2^). Because the correlation between finger patterns within a single condition is captured in the strength of the pattern *u*_*α*[*c*]_, and for patterns of different fingers across condition by *γ*_*α*_, these pattern components are uncorrelated. Only patterns for matching fingers share the additional covariance *γ*_*β*_. It is these parameters that will tell us how much finger-specific variance or information is shared across conditions. In sum, our covariance component model is:

(9) $$G=\text{var}\left[\begin{array}{c} u_{\alpha [1]}\\ u_{\alpha [2]}\\ u_{\beta [1,1]}\\ ...\\ u_{\beta [1,4]}\\ u_{\beta [2,1]}\\ ...\\ u_{\beta [2,4]} \end{array}\right]=\left[\begin{array}{cccccccc} \sigma_{\alpha [1]}^{2} & \gamma_{\alpha } & 0 & \cdots & 0 & 0 & \cdots & 0\\ \gamma_{\alpha } & \sigma_{\alpha [2]}^{2} & 0 & \cdots & 0 & 0 & \cdots & 0\\ 0 & 0 & \sigma_{\beta [1]}^{2} & & 0 & \gamma_{\beta } & & 0\\ \vdots & \vdots & & \ddots & & & \ddots & \\ 0 & 0 & 0 & & \sigma_{\beta [1]}^{2} & 0 & & \gamma_{\beta }\\ 0 & 0 & \gamma_{\beta } & & 0 & \sigma_{\beta [2]}^{2} & & 0\\ \vdots & \vdots & & \ddots & & & \ddots & \\ 0 & 0 & 0 & & \gamma_{\beta } & 0 & & \sigma_{\beta [2]}^{2} \end{array}\right]$$

Under this model, the expected value of the sample correlation between the measured patterns for the same finger for the movement and stimulation condition is:

(10) $$E\left(\text{cor}\left({\bar{y}}_{1,i},{\bar{y}}_{2,i}\right)\right)=\frac{\gamma_{\alpha }+\gamma_{\beta }}{\sqrt{\left(\sigma_{\alpha [1]}^{2}+\sigma_{\beta [1]}^{2}+\sigma_{\varepsilon }^{2}/n\right)\left(\sigma_{\alpha [2]}^{2}+\sigma_{\beta [2]}^{2}+\sigma_{\varepsilon }^{2}/n\right)}}$$

Whereas the sample correlation between non-matching fingers would be

(11) $$E\left(\text{cor}{\left({\bar{y}}_{1,i},{\bar{y}}_{2,j}\right)}_{i\neq j}\right)=\frac{\gamma_{\alpha }}{\sqrt{\left(\sigma_{\alpha [1]}^{2}+\sigma_{\beta [1]}^{2}+\sigma_{\varepsilon }^{2}/n\right)\left(\sigma_{\alpha [2]}^{2}+\sigma_{\beta [2]}^{2}+\sigma_{\varepsilon }^{2}/n\right)}}$$

**Fig. 4:**
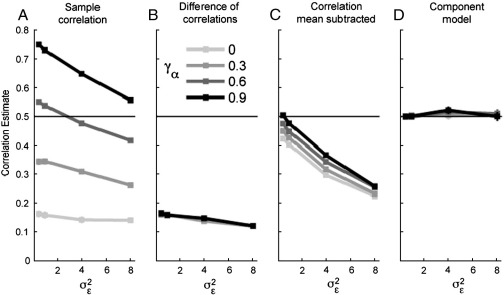
Estimates for the representational similarity between sensory and motor representations of the same finger (true value *r* = 0.5), as a function of the level of noise (*σ*_*ε*_^2^) and the covariance of the patterns common to the movement and stimulation conditions (*γ*_*α*_). (A) The sample correlation calculated on the mean activation patterns for identical fingers across conditions is strongly influenced by noise and common activation. (B) By subtracting the correlation across conditions for different fingers, the influence of the common activation is eliminated. However, the correlation is underestimated and biased (downwards) by noise. (C) The correlation between patterns for the same fingers, after subtracting the mean pattern for the respective condition accounts partly for the effect of common activation, but is severely biased by noise. (D) The corrected estimate from the covariance-component model is unbiased over a large range of parameter settings.

As we can see, these sample correlations are influenced by many factors other than the true similarity *γ*_*β*_. We simulated data with the parameters *σ*_*β*[1]_^2^ = *σ*_*β*[2]_^2^ = 1, *σ*_*α*[1]_^2^ = *σ*_*α*[2]_^2^ = 2, *γ*_*β*_ = 0.5, and varied the two parameters *σ*_*ε*_^2^ ∈ {0.5,..., 8} and *γ*_*α*_/*σ*_*α*_^2^ ∈ {0,..., 0.9}. The sample correlation between matching fingers (Fig. 4A) was influenced by both of these factors: as the noise-level increased, the correlation dropped. Furthermore, as the true nonspecific correlation (*γ*_*α*_/*σ*_*α*_^2^) between the activations increased, so did the sample correlations. This makes it very difficult to compare sample correlations across regions or groups of participants.

An alternative strategy is to compare the correlations between movement and stimulation of the same finger to the correlations between different fingers. This analysis removes the dependency on *γ*_*α*_ (Fig. 4B). However, the difference between correlations underestimates the true correlation between finger patterns and still depends on the noise level.

Third, as considered in the one-factorial design, one could estimate the nonspecific condition effect by calculating the mean patterns for all trials of one condition. One could then subtract this pattern from all finger-specific patterns of the same condition and then examine the correlations between the residual patterns. This represents an ad-hoc attempt to decompose the patterns into common and specific components. However, this fixed-effects approach does not recognize that the sample mean of all patterns (common mean) also contains noise. In Correcting for a common activation pattern we had seen how the subtraction of a pattern estimated from an independent control condition induces an artificial positive correlation between patterns. In this case we would subtract the mean over the conditions and thereby induce an artificial negative correlation between the residuals. The correlation between patterns of different conditions therefore decreases with increasing noise (Fig. 4C), which again makes it impossible to compare correlations from different regions.

Thus, as we have seen before, there is no simple ‘fix’ for sample correlations that would enable them to be compared meaningfully. Our model solves this problem by estimating explicitly the different covariance components in Eq. (9). By doing this, we obtain the corrected correlation *γ*_*β*_/*σ*_*β*[1]_*σ*_*β*[2]_. This estimate (see Fig. 4D) is stable across variations in the amplitude of noise or the nonspecific component. As such, this corrected correlation provides a robust measure of pattern similarity that can be compared meaningfully across different regions or participants.

### The influence of voxel selection

A further factor that influences the sample correlation is the composition of the region's voxels. While our model assumes that the pattern components will have an average variance across different voxels, it is very unlikely to pick voxels in which the patterns are represented homogenously. If the region that we pick contains informative voxels for half, and non-informative voxels for the other half, the correlation will be lower than when the region contains mostly informative voxels.

We tested whether the pattern component model can deal with this problem. For this simulation, we used the two-factorial 2x4 (movement vs. stimulation) design described in the previous section. For one portion of the voxels we set the simulation values to *σ*_*ε*_^2^ = 4, *σ*_*α*[1]_^2^ = *σ*_*α*[2]_^2^ = 2 and *σ*_*β*[1]_^2^ = *σ*_*β*[2]_^2^ = 1, *γ*_*α*_ = 0, and *γ*_*β*_ = 0.5. For a subset of voxels (varying between 0% and 75%), we set *σ*_*β*_^2^ to zero; i.e., these voxels did not contain information about the finger involved.

**Fig. 5:**
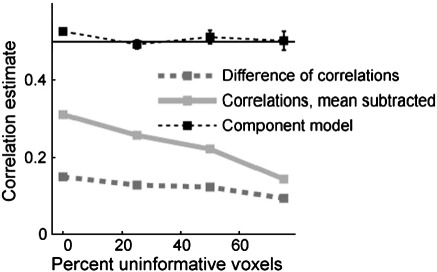
Correlation estimate (true value *r* = 0.5) changes with increasing numbers of non-informative voxels. This makes it impossible to compare correlations across different regions. The graph shows the difference between sample correlation for same and different finger (dark gray dashed line) and the correlation between the patterns after subtracting the corresponding condition mean (light gray). The corrected estimate from the pattern-component model (black dashed) remains valid, even if 75% of the voxels in the studied regions are uninformative.

As can be seen from Fig. 5, increasing the number of non-informative voxels in the region of interest has the same effect as increasing noise: The mean-corrected sample correlation or the difference between sample correlations declines with the number of informative voxels. In contrast, the correlation estimate from the covariance component model *r*_*β*_ = *γ*_*β*_/*σ*_*β*1_*σ*_*β*2_ retains its unbiased behaviour. This is because both the estimates for *σ*_*β*_^2^ and *γ*_*β*_ decline simultaneously with the number of informative voxels.

### Similarity of representations across conditions: real data example

**Fig. 6:**
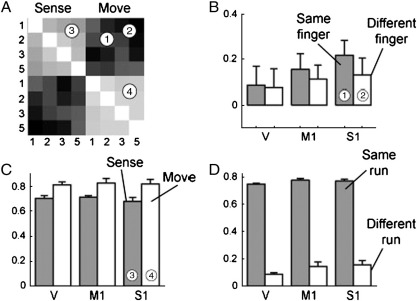
Traditional representational similarity analysis using sample correlations from a real data set. (A) Correlation-matrix for primary somatosensory cortex (S1) for two conditions (sense and move) and four levels (fingers 1, 2, 3 and 5). Bright squares indicate high correlations, dark squares zero or slight negative correlations. (B) In S1, the average correlation between movement and sensory patterns for the same finger (1) was elevated compared to those between different fingers (2). This was also the case for M1, but not for lobule V of the cerebellum. (C) The correlations between patterns for different fingers within the same condition were higher and more pronounced in the movement (4) than in the sensory (3) condition, suggesting different strength of the common activation patterns. (D) These correlations were much higher for patterns estimated within the same run than for different runs, indicating a strong covariance in the estimation errors. Error bars indicate between-subject (*N* = 7) standard error.

Having established the robustness of our approach, we now turn to a real data example. The design of the experiment (Wiestler et al., 2009) is described in the previous section. The main focus of this experiment was to compare the similarity of sensory and motor representations of fingers in the cerebellum (lobule V) and the neocortex (primary somatosensory cortex, S1, and primary motor cortex, M1). Fig. 6 shows the results of a traditional representational similarity analysis. Here, we calculated the sample correlations between the mean patterns for each finger (digits 1, 2, 3, and 5) and condition (sense vs. move) to obtain an 8 × 8 correlation matrix (Fig. 6A). The patterns for moving a specific finger correlated with the pattern for stimulation of the same finger (1). To determine whether this similarity was finger-specific, or whether it was caused by a nonspecific similarity between motor and sensory patterns, we compared these correlations to those calculated across conditions for the six possible pairings of different fingers (2). An interesting effect was found in the between-region comparison (Fig. 6B): in the neocortex the correlation between patterns of movement and sensory stimulation for the same finger was higher than for different fingers, while in the cerebellum no such difference was found. This result, however, needs to be considered with caution, because, as seen above, differences between sample correlations from different regions cannot be compared. Because the correlations in the cerebellum were roughly half the size compared to those in the neocortex, it seems likely that the variance of the noise component was higher here.

Before calculating corrected correlations using our method, we need to consider a further detail: When we looked at the correlations between different fingers within the sensory and movement conditions (Fig. 6C), we found these correlations to be very high in both regions. In the experiment we blocked the conditions to simplify instructions to the participant. In the first half of each run, participants performed trials in one condition, followed by the other condition in the second half, interrupted by a relatively short resting period. Because of this, the estimation errors of the regression coefficients will be correlated within each run and condition, while they should be uncorrelated across runs or conditions. Indeed, when we calculated the average sample correlations between the patterns of each run (different fingers, same condition), we found that these correlations were substantially higher than the same correlations calculated across runs (Fig. 6D). This finding shows that correlation between patterns can also be increased by noise due to conditional dependencies among estimators from a single run, and underscores the importance of performing cross-validation across different imaging runs.

The decomposition method offers an elegant way to control for all these possible influences on the size of the correlation coefficients in a single modelling framework. In addition to noise (*ε*), condition (*u*_*α*[1]_,*u*_*α*[2]_), and finger (*u*_*β*[1]_,*u*_*β*[2]_) effects (Eq. (8)), we also added a run effect. This pattern component was common to all trials of one condition within the same run, but uncorrelated across runs. Thus, we allowed separate pattern components for each run (indexed by *i*, *u*_*δ*[1, *i*]_,*u*_*δ*[2, *i*]_), and estimated separate variances for the two conditions (*σ*_*δ*[1]_^2^ vs. *σ*_*δ*[2]_^2^) and their covariance (*γ*_*δ*_) within a run.

**Fig. 7:**
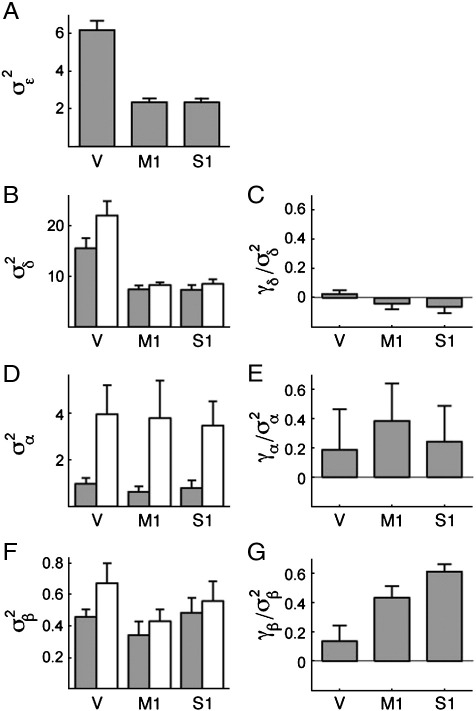
Decomposition of the correlations shown in Fig. 6 into pattern components. (A) The estimated variance of the noise component (*σ*_*ε*_^2^) was 2.5 times stronger for the cerebellar lobule V than for the primary motor cortex (M1) and primary sensory cortex (S1). (B) The effect of run (*σ*_*δ*_^2^) was strong for both sensory (gray) and movement (white) condition and scaled in the same way as the noise component. (C) The effect was uncorrelated across conditions within the same run. (D) The variance of the common condition component (*σ*_*α*_^2^) was stronger for the movement (white) than for the sensory (gray) condition. (E) The components for the two conditions were slightly correlated. (F) The variance of the finger component (*σ*_*β*_^2^) was roughly matched across regions. (G) Covariance of the finger components across the two conditions confirms that there is a difference in the organization of sensory and motor maps between neocortex and cerebellum. Error bars indicate between-subject (*N* = 7) standard error.

Fig. 7 shows the decomposition into the different components. The noise variance (Fig. 7A) was indeed substantially higher in the cerebellum compared to the neocortex (by a factor of 2.5), which emphasizes the importance of accounting for noise when comparing correlations. The run effect (Fig. 7B), caused by correlated estimation errors, showed a similar difference between cerebellum and neocortex, consistent with the idea that the covariance was induced by noise in the estimation of the common resting baseline. The correlation coefficient (*γ*_*δ*_/*σ*_*δ*[1]_*σ*_*δ*[2]_, Fig. 7C) also showed that the run effect was uncorrelated across conditions. This makes sense as the two conditions were acquired in two different halves of each run, making their estimation nearly independent.

Having accounted for the noise components, we can now investigate the condition effect (*σ*_*α*_, Fig. 7D, E), which is common to all fingers. These pattern components were much stronger in the movement condition, consistent with the observation that the BOLD signal changes much more during the movement compared to the stimulation condition. In contrast, the variance of the components that were unique to each finger (*σ*_*β*_, Fig. 7F) was similar across conditions and regions.

Of key interest, however, are the corrected correlation coefficients (similarity indices) between the motor and sensory patterns for the same finger (*γ*_*δ*_/*σ*_*δ*[1]_*σ*_*δ*[2]_, Fig. 7G). These indices are significantly different between cerebellum and neocortical regions (paired *t*-test for *N* = 7 participants, *t*(6) = −4.09, *p* = 0.006), arguing strongly that the difference in correlation structure observed in Fig. 6B was caused by a difference in the neural representation in these regions, and not by an effect of noise, voxel selection, or covariance in estimation (Wiestler et al., 2009).

## Covariance between voxels

So far, we have looked at the covariance structure of the data over trials, ignoring the possible spatial dependence of voxels. Even in unsmoothed fMRI data, however, spatial correlations clearly exist, and may contain valuable information about the spatial structure of the underlying representations. Although the full integration of spatial covariances into the model is beyond the scope of this paper, we sketch out here how such correlations would be incorporated. We will then test, with simulated data, how spatial correlations influence our estimates of variance–covariance structure of the hidden pattern components. Finally, we will suggest a simple method to estimate the spatial smoothness of each of the pattern components, allowing some insight into their spatial structure.

To include spatial smoothness into our model, we need to specify the correlation structure of the matrix **U** not only between the hidden patterns, but also between voxels or features. We can do this by specifying the variance of a row of **U** to be $\text{var}\left(u_{i}^{r}\right)=\sum g_{i,i}$, where ∑ is a *PxP* covariance matrix that determines the distribution of the pattern component across voxels, and *g*_*i*, *i*_ is the *i*-th element of the diagonal of **G**, indicating the variance of this component. To avoid redundancy, we assume the mean of the diagonal elements of *Σ*is 1.

Now we have to deal both with covariance across trials and covariance across voxels at the same time. To be able to write the full covariance structure, we need to rearrange our data matrix **Y** into a *NxP* vector by stacking the rows (via the vec() operator). Similarly we stack the rows of **U**, such that we obtain an *NxQ* vector. The new variance–covariance matrix $\tilde{G}$ (now a (NxQ)x(NxQ) matrix) can be written using the Kronecker tensor product: $\tilde{G}=\text{var}\left(\text{vec}\left(U\right)\right)=G⊗\sum$.

We now can allow each pattern component to have its own spatial covariance structure. For example, we may hypothesize that the activation pattern elicited by the overall task of moving a finger ($u_{\alpha }$ in above example) is relatively smooth, while the patterns specific to the individual fingers ($u_{\beta }$) maybe more fractionated. Thus, we can partition the rows of **U** into *J* sets, each of which is associated with its own spatial covariance kernel *Σ*_*j*_. Here, we assume that these subsets correspond to different diagonal blocks of **G** (**G**_**1**_**, G**_**2**_, …). Under this formalism, the covariance matrix becomes:

(12) $$\tilde{G}=\text{var}(\text{vec}(U))=\left[\begin{array}{ccc} G_{1}⊗\sum_{1} & 0 & \\ 0 & G_{2}⊗\sum_{2} & \\ & & \ddots \end{array}\right]$$

Finally we posit that the noise, which is independent over the trials, has its own spatial covariance matrix ∑_*ε*_.

(13) $$\text{var}(\text{vec}(E))=I_{N}⊗\sum_{\varepsilon }$$

### Influence on the estimation of **G**

If different spatial smoothness for different components are a reality – and all our experience so far indicates that this is the case – then we have to first worry about how this would influence the estimation of the elements of **G**. We expected a priori that smoothness should not bias the estimation of variance, but only its precision (the degrees of freedom of the variance estimator). To test this assumption we simulated data using the 4 × 2 design described in Accessing similarities across conditions. We then introduced a spatial smoothness between neighbouring voxels that decayed exponentially with the square of the distance *δ* between two voxels.

(14) $$\text{corr}\left(u_{i}\left(x\right),u_{i}\left(x+\delta \right)\right)=\text{exp}\left(-\frac{\delta^{2}}{2s_{i}^{2}}\right)$$

where *s*_*i*_ indicates the standard deviation of the spatial autocorrelation function. If *s* is small, neighbouring voxels will be relatively independent. The smoothness can also be expressed as the FWHM of the Gaussian smoothing kernel that – applied to spatially independent data – would give rise to the same spatial autocorrelation function. The SD of this kernel is $\sqrt{1/2}s_{i}$ , and its FWHM can be calculated as:

(15) $${\text{FWHM}}_{i}=2\sqrt{\text{log}\left(2\right)}\;s_{i}\text{.}$$

We used different spatial kernels for the overall condition effect (*α*), the effect of finger (*β*) and the noise patterns (*ε*). We simulated a sphere of 160 voxels (3.5 voxels radius) with the parameter values *σ*_*ε*_^2^ = 3, *σ*_*α*1_^2^ = *σ*_*α*2_^2^ = 1 and *σ*_*β*1_^2^ = *σ*_*β*2_^2^ = 1, *γ*_*α*_ = 0.3, *γ*_*β*_ = 0.5, and varied the spatial kernels, with *s*_*β*_ ∈ {0, 1, 2}, and *s*_*ε*_^2^ ∈ {0, 1, 2}, while leaving *s*_*α*_ = 1.

**Fig. 8:**
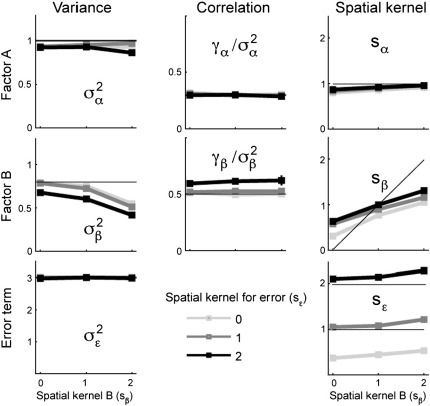
Stability of the estimators with respect to spatial smoothness of the pattern component of Factor B (SD of autocorrelation function,*s*_*β*_, *x*-axis) and the noise component (*s*_*ε*_, different lines). The first column shows the variance estimates, and second correlation estimates for the experimental factors A and B. The last column shows the estimates for the SD parameter of the spatial autocorrelation function (see text for details).

The results of this simulation are shown in Fig. 8. The estimates of the variances (left column) and the correlation (middle column) for the two factors and for the error term remain relatively stable. Only the variance estimates for the weakest effect (*σ*_*β*_^2^) are biased downward, when the FWHM (*s* = 2 corresponds to a FWHM of 3.33 voxels) approaches the radius of the search region. However, for a search region with a diameter of 7 voxels and FWHM below 3 voxels, the estimates remain reassuringly stable.

### Estimating the width of covariance kernels

Would covariance partitioning allow us to estimate the width of the covariance kernel for the experimental factors in question? Such an estimate would relate to the size of the neural clusters that show similar BOLD responses for the component in question. For example, nonspecific activations related to the task may be relatively smooth and cover large regions, while the pattern components distinguishing individual fingers may be much more fine-grained. So can we recover this spatial information for each component?

The Kronecker form of the generative model (Eqs. (12) and (13)) makes its inversion very slow. Alternatively one can employ an approximate two-step procedure, by first estimating the variance–covariance structure among components, ignoring any spatial dependence, and then obtaining a simple estimate for the spatial covariances, based on the current estimates of the hidden patterns, **U** (from Eq. (4)). To do this, we calculated the sample autocorrelation function (over voxels) using the appropriate rows of **U** (within all levels of a particular factor). To summarize these empirical estimates, we then determined *s* by fitting an exponential kernel (Eq. (14)).

The resulting estimates are shown in the third column for Fig. 8 for the simulated data above. Whereas the estimates for *s*_*α*_ and *s*_*ε*_ are relatively near to the true values (indicated by lines), the estimates for the weakest effect, *β*, are somewhat biased by the other values. First, for true values of *s*_*β*_ of 0 and 2, the estimates are biased towards the value of *s*_*α*_^2^(1). Furthermore, the spatial smoothness of the noise effect also influences the estimates.

These biases reflect the fact that simply estimating the sample autocorrelation function provides suboptimal estimates. However, optimum estimators are rather difficult to obtain. We would need to specify our Gaussian process model in terms of vectorised responses (as above), because the covariance structure cannot be factorized into spatial and non-spatial (experimental) factors. This somewhat destroys the efficiency and utility of Gaussian process modelling of multivariate responses. Our simulation, however, demonstrates that even with our approximate method, we can obtain an estimate of the spatial smoothness for each component.

### Estimating the width of covariance kernels: real data example

To illustrate the utility of this method, we applied it to the data described in Similarity of representations across conditions: real data example. For primary sensory and motor cortex, and for the hand area in lobule V, we decomposed the covariance kernels separately for the condition, finger, run and noise effect. Based on the final estimate of **U**, we calculated the autocorrelation function for over 11 spatial bins, ranging from 0.1–2.5 mm (directly neighbouring), 2.5–3.6 mm (diagonally neighbouring), up to a total distance of 23.8 mm. To summarize the autocorrelation functions, we fitted a squared-exponential kernel (Eq. (14)) to the sample autocorrelation functions.

**Fig. 9:**
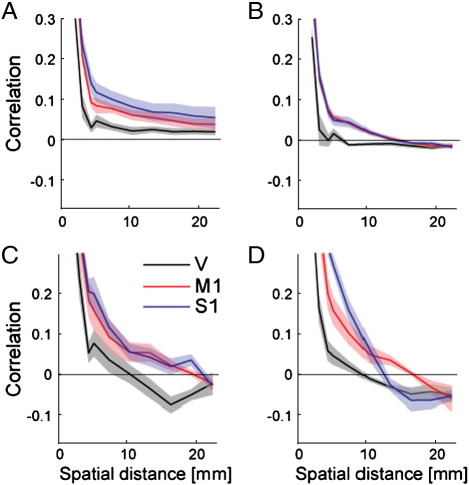
Estimates of the spatial auto-correlation from 4 different pattern components for primary sensory cortex (S1), primary motor cortex (M1) and lobule V of the cerebellum (V). The noise components (A) and run component (B) show rapidly decaying spatial autocorrelation functions, with slightly wider correlations in cortical than in cerebellar regions. (C) The main effect of condition shows wider correlation kernels, indicating that larger groups of voxels increase or decrease consistently in movement and stimulation condition. (D) The effect of finger indicates slightly larger finger patches in S1 than in M1, with the representations in lobule V being smaller than the effective resolution.

The autocorrelation functions for the noise component (Fig. 9A) and for the run component (Fig. 9B) were very similar, with an average FWHM of 2.17 mm for the cerebellum and 2.9 mm for neocortical regions, *t*(6) = 6.43, *p* = .001. The similarity of the spatial structure of these two components agrees with the hypothesis that both result from similar noise processes. The results also show that noise has a spatial smoothness roughly one voxel (2 mm).

In contrast, the effects for condition (Fig. 9C) and finger (Fig. 9D) have a significantly greater smoothness, both for lobules V, *t*(6) = 3.87, *p* = .008, as well as for the two cortical areas; both *t*(6) > 4.46, *p* = .004. This indicates that the spatial scales of different pattern components can be different and that our method can (albeit imperfectly) detect these differences.

Interestingly, we found a difference in the estimated size of the finger representation: We estimated the FWHM for S1 to be 5.1 mm, and for M1 to be 4.1 mm a significant difference, *t*(6) = 4.34, *p* = .027. Note that in the other components, no differences were found between these two regions. In the cerebellum, the representation was smaller again with a FWHM of 2.3 mm, *t*(6) = 5.83 *p* = .001. Thus, these results are consistent with the known characteristics of somatosensory representations in the neocortex and cerebellum (see Wiestler et al., 2009).

## Outlook and conclusion

The current algorithm and formulation furnishes estimates of the true similarity of patterns of distributed responses for subsequent analysis. We have focused here on correlation coefficients as similarity measures. The same covariance estimates could also be used to provide corrected estimates for the Euclidian distance between patterns. Technically, the innovative step presented in this paper is to parameterize **G** as **AA**^**T**^, which renders the problem linear in the hyper-parameters (see also Wipf and Nagarajan, 2009).

We anticipate that this approach could be extended in two directions. First, our model could be used to compare different covariance models using the marginal likelihood $p\left(Y|m\right)$. For this we would have to impose priors on the free parameters, effectively changing the EM-scheme into Variational Bayes. Imposing priors may also address a problem of stability in the current formulation, in that the variance of the normalized correlation coefficients (Eq. (6) becomes large, as the variance of the pattern *σ*^2^ becomes small. In the current approach, the user needs to ensure that the variances are sufficiently large, and use a simpler model when the region does not encode the factor in question. The use of priors (and marginal likelihoods or model evidence) would enable us to use automatic relevance detection to automatically drop terms from the model that do not help to explain the data.

A second extension is to include and explicitly estimate the spatial parameters of the underlying patterns. This would unify this approach with a multivariate Bayesian approach to pattern analysis, in which only the correlation structure between voxels, but not between trials or conditions, is parameterized (Friston et al., 2008).

In its current implementation, our algorithm provides a concise way of estimating the similarity of multivariate patterns, and enables researchers to compare these measures directly between different regions and brains.

## Appendix A EM-Algorithm to estimate covariance matrices with linear constraints on its factors

### The model

The algorithm presented here provides inference on covariance component models, in which linear constraints are placed on factors of the variance–covariance matrix. Each of the *N* observations (referring to time points or trials) $y_{n}^{r}$ is a *Px1* vector. The data therefore comprise an *NxP* matrix (see Fig. 1). We model observed covariances as a mixture of *Q* hidden pattern components encoded in a *QxP* pattern matrix **U**. The *P* columns ($u_{p}^{c}$) of **U** are randomly distributed over voxels:

(A1) $$\begin{array}{c} y_{p}^{c}=Zu_{p}^{c}+\varepsilon_{p}^{c}\\ u_{p}^{c}\sim N\left(0,G\right)\\ \varepsilon_{p}^{c}\sim N\left(0,I\sigma_{\varepsilon }^{2}\right) \end{array}$$

This is a standard random effects model. If the variance–covariance matrix **G** is completely unconstrained, the EM algorithm proposed by Laird et al. (1987) would provide an efficient solution for estimation. However, in many cases we would like to estimate the variances or covariances under certain structural assumptions. For example, we may not be interested in the variance attributed to each stimulus, but may be interested in the average variance that encodes a certain factor. That is, we would like to assume that the variance of all levels within that factor is the same. This could be done by expressing **G** as a linear mixture of *K* components, each weighted by an unknown parameter *θ*_*k*_

(A2) $$G=\sum_{k}^{}\theta_{k}G_{k}$$

Such parameterizations can be estimated using a Newton-Raphson algorithm (Friston, 2008). The main problem in such a scheme, however, is to ensure that **G** remains positive-definite. If the components of **G** are diagonal, a positive-definite **G** can be enforced by estimating the log of *θ*, thereby ensuring *θ* > 0. For situations in which we also wish to estimate covariances, such a scheme sometimes fails, because it does not enforce the Cauchy-Schwarz inequality $\left|\gamma_{\mathrm{ij}}\right|<\sqrt{\sigma_{i}^{2}\sigma_{j}^{2}}$. One can try to address this by rewriting **Z** and **G**, such that the covariances between conditions are captured by the summation over separate, independent factors. Because **G** now again has a diagonal form, positive-definiteness can easily be ensured. This formulation, however, restricts the covariance estimate by *γ* > 0 and *γ* < min(*σ*_1_^2^, *σ*_2_^2^), rather than enforcing the more flexible Cauchy–Schwarz inequality.

To solve this problem, we impose constraints on the square root (factor) of the covariance matrix and estimate its parameters.

(A3) $$\begin{array}{c} G=AA^{T}\\ A=\sum_{k}^{}\theta_{k}A_{k} \end{array}$$

The (matrix) squaring ensures that **G** is positive semi-definite for any **A**, and the addition of positive *σ*_*ε*_^2^ then ensures the overall covariance $ZGZ^{T}+\sigma_{\varepsilon }^{2}I$ is positive definite.

### Linear constraints

Eq. (A3) allows us to enforce independence constraints (elements of **G** that need to be 0) and equality constraints (elements of **G** that need to be equal). In general, many structurally equivalent parameterizations of A for each desired structure of **G** are possible (Pinheiro and Bates, 1995). In the following we will give a number of examples for different types of constraints. In the simple case of an unconstrained example for a 2 × 2 covariance matrix, we could use the following elements of **A**:

(A4) $$G=\left[\begin{array}{cc} \sigma_{1}^{2} & \gamma \\ \gamma & \sigma_{2}^{2} \end{array}\right]\to A_{k}=\left\{\left[\begin{array}{cc} 1 & 0\\ 0 & 0 \end{array}\right],\left[\begin{array}{cc} 0 & 0\\ 0 & 1 \end{array}\right],\left[\begin{array}{cc} 1 & 1\\ 1 & 1 \end{array}\right]\right\}$$

If we want to enforce equality of the diagonal elements (variances), one less element for **A** is used.

(A5) $$G=\left[\begin{array}{cc} \sigma^{2} & \gamma \\ \gamma & \sigma^{2} \end{array}\right]\to A_{k}=\left\{\left[\begin{array}{cc} 1 & 0\\ 0 & 1 \end{array}\right],\left[\begin{array}{cc} 1 & 1\\ 1 & 1 \end{array}\right]\right\}$$

If **G** has a block-diagonal structure, then all elements of **A** ($A_{k}$) also need to share the same block-diagonal structure. For example the variance–covariance matrix in Eq. (9) can be rearranged in a block-diagonal form by swapping rows and columns. We can then enforce equality constraints across different blocks by having joint elements for each of the blocks.

(A6) $$G=\left[\begin{array}{cccc} \sigma^{2} & \gamma & 0 & 0\\ \gamma & \sigma^{2} & 0 & 0\\ 0 & 0 & \sigma^{2} & \gamma \\ 0 & 0 & \gamma & \sigma^{2} \end{array}\right]\to A_{k}=\left\{\left[\begin{array}{cccc} 1 & 0 & 0 & 0\\ 0 & 1 & 0 & 0\\ 0 & 0 & 1 & 0\\ 0 & 0 & 0 & 1 \end{array}\right],\left[\begin{array}{cccc} 1 & 1 & 0 & 0\\ 1 & 1 & 0 & 0\\ 0 & 0 & 1 & 1\\ 0 & 0 & 1 & 1 \end{array}\right]\right\}$$

However, it should be noted that it is not possible to enforce equal covariances across different blocks, while allowing different variances. Similarly, it is also not possible to enforce equal variances, while allowing different covariances. Thus, the variance–covariance structures of the block over which we want to enforce equality constraints need to be identical.

One last example considers an unconstrained estimation of the elements of **G**, when one of the pairs of stimuli is uncorrelated. This situation may arise when we want to estimate the similarity structure between a set of stimuli and would like to remove the common task activation by introducing a common pattern. To disambiguate the common activation and correlation of individual patterns, the correlation of one pair of stimuli (here between 1 and 3) needs to be held constant.

(A7) $$G=\left[\begin{array}{ccc} \sigma_{1}^{2} & \gamma_{1,2} & 0\\ \gamma_{2,1} & \sigma_{2}^{2} & \gamma_{2,3}\\ 0 & \gamma_{2,3} & \sigma_{3}^{2} \end{array}\right]$$

To enforce such a constraint, we would have *k* = 5 basis matrices, in which the position of the corresponding parameter in the lower triangular part of **G** is set to 1. A linear combination of these basis matrices then forms **G**'s Cholesky factor.

### Limitation on the possible structure of independence constraints

In general, independence constraints between pattern components are encoded in the sparsity patterns of **G**. It is important to note that not all sparsity patterns of **G** can be translated into corresponding sparsity patterns of the Cholesky factors. For example, the solution employed for Eq. (A7) would not work if we try to set *γ*_2, 3_ to zero and allow *γ*_1, 3_ to be non-zero. In this case, a fill-in occurs; the Cholesky factor has six non-zero elements.

Thus, for some independence structures, we need to re-order the rows and columns. For most cases, MATLAB's chol command for sparse matrices can be used with multiple return arguments, such that it permutes the rows and columns to obtain an appropriate decomposition. However, some structures produce an unavoidable fill-in, even allowing for permutations. One of the simplest examples is given by the sparsity pattern *S* = toeplitz([1 1 0 1]). Regardless of the values of the 8 unique nonzero elements, the Cholesky factorization has 9 values, meaning that this square root parameterization would have some redundancy.

There is an interesting connection here to graph theory: Parter (1961) established a link between Gaussian elimination (from which Cholesky factorization emerges for symmetric positive definite matrices) and eliminating vertices from a graph with adjacency matrix given by the nonzero non-diagonal elements of the original matrix. Fill-in corresponds to edges that must be added to make the neighbourhood of a candidate vertex into a clique before removing that vertex and its edges. Rose (1970), characterized graphs allowing a perfect (zero fill-in) elimination ordering of their vertices as “chordal,” i.e. having no cycles of length 4 or more without a chord joining a pair of non-consecutive vertices. The problematic sparsity pattern S given above corresponds to the simplest such graph: a square, in which one may form a cycle from element 1 to 2 to 3 to 4 and back to 1. Adding a chord across either diagonal of the square, corresponding to matrix elements (1, 3) or (2, 4), allows a perfect elimination ordering to be found.

For most desired similarity structures of **G**, however, a matching similarity structure in **A** can be found. For some examples, one needs to rearrange rows and columns to avoid redundancy in the parameterization. For chordal graphs a perfect ordering can be found using maximum cardinality search (Berry et al., 2009). For the rare similarity structures that correspond to non-chordal graphs, there will be some redundancy in their Cholesky factors. Minimizing this redundancy is NP-hard, but approximate solutions can be efficiently found using a minimum degree heuristic (Berry et al., 2003), as available in MATLAB's chol command.

### Estimating the variance–covariance structure

After defining possible constraints, we want to estimate *θ* by optimizing the likelihood $p\left(Y|\theta \right)$. This optimization problem can be transformed into a regression problem, by introducing a new set of hidden variables **v** that have i.i.d. multivariate normal distribution, with identity covariance. In the following all **y**, **v**, **u**, and *ε* are Nx1 column vectors for each voxel, we will drop the superscript *c*.

(A8) $$\begin{array}{l} u_{p}^{}=Av_{p}\\ \text{var}\left(u_{p}^{}\right)=A\text{var}\left(v_{p}^{}\right)A^{T}=AA^{T} \end{array}$$

We can now replace **ZA** with a new variable **C** and effectively replace the constraints in Eq. (A3) with constraints on **C**.

(A9) $$\begin{array}{c} y_{p}^{}=ZAv_{p}+\varepsilon_{p}≜Cv_{p}+\varepsilon_{p}\\ C=\sum_{k}^{}\theta_{k}ZA_{k}=\sum_{k}^{}\theta_{k}C_{k}\\ \varepsilon_{p}\sim N\left(0,I\theta_{\varepsilon }\right)\\ v_{p}\sim N\left(0,I\right) \end{array}$$

This model is equivalent to stochastic factor analysis with constraints imposed on the loading matrix **C**. The complete log-likelihood of both the data **Y** and the hidden variables **V** together is:

(A10) $$\begin{array}{c} \text{log}p_{c}\left(Y,V|\theta ,\sigma_{\varepsilon }^{2}\right)=-\frac{N}{2}\text{log}\left|I\sigma_{\varepsilon }^{2}\right|-\frac{1}{2}\sum_{p}v_{p}^{T}v_{p}-\frac{1}{2}\sum_{p}{\left(y_{p}-Cv_{p}\right)}^{T}\sigma_{\varepsilon }^{-2}\left(y_{p}-Cv_{p}\right)\\ =-\frac{N}{2}\text{log}\left|I\sigma_{\varepsilon }^{2}\right|-\frac{1}{2}\sum_{p}v_{p}^{T}v_{p}-\frac{1}{2}\sum_{p}tr\left(\left(y_{p}-Cv_{p}\right){\left(y_{p}-Cv_{p}\right)}^{T}\sigma_{\varepsilon }^{-2}\right)\\ =-\frac{N}{2}\text{log}\left|I\sigma_{\varepsilon }^{2}\right|-\frac{1}{2}\sum_{p}v_{p}^{T}v_{p}-\frac{1}{2}\sum_{p}tr\left(\left(y_{p}y_{p}^{T}-y_{p}v_{p}^{T}C^{T}-Cv_{p}^{}y_{p}^{T}+Cv_{p}v_{p}^{T}C^{T}\right)\sigma_{\varepsilon }^{-2}\right) \end{array}$$

To estimate *θ* and *σ*_*ε*_^2^ we would have to integrate out the hidden parameters **v**, $p\left(Y|\theta \right)=\int p_{c}\left(Y,|V,\theta ,\sigma_{\varepsilon }^{2}\right)p\left(V\right)dV$, and maximize this quantity. Because no closed form for this integral exists, we use the Expectation-Maximization algorithm (McLachlan, 1997) to instead maximize a lower bound on the log-likelihood, the free energy *F*.

(A11) $$\begin{array}{l} \text{log}\int p_{c}\left(Y,V|\theta ,\sigma_{{}^{\varepsilon }}^{2}\right)dV\geq \int q\left(V\right)\text{log}\left(\frac{p_{c}\left(Y,V|\theta ,\sigma_{\varepsilon }^{2}\right)}{q\left(V\right)}\right)dV\\ ={\langle \text{log}p_{c}\left(Y,V|\theta ,\sigma_{\varepsilon }^{2}\right)\rangle }_{q}-{\langle \text{log}q\left(V\right)\rangle }_{q}=F\left(Y,V|\theta ,\sigma_{\varepsilon }^{2}\right) \end{array}$$

Where 〈〉_*q*_ is the expected value under the proposal distribution. It can be shown that if $q\left(v\right)$ is the posterior distribution over **V** given the parameters, the bound becomes tight and *F* becomes the log-likelihood that we are attempting to optimize.

Thus, in the E-step we calculate the posterior distribution of **V**, given the current estimate of *θ* and *σ*_*ε*_^2^. Because the noise is normally distributed, the posterior distribution over **V** also has multivariate normal distribution, meaning we only have to calculate the posterior mean and variance. On the first E-step we start with an initial guess on the parameters. Each iteration u then follows as:

$$C=\sum_{k=1}^{K}\theta_{k}^{\left(u\right)}C_{k}$$

(A12) $$\begin{array}{c} \text{var}\left(y_{p}\right)=I\sigma_{\varepsilon }^{2\left(u\right)}+CC^{T}\\ \langle v_{p}\rangle =C^{T}\text{var}{\left(y_{p}\right)}^{-1}y_{p}\\ \text{var}\left(v_{p}|y_{p}\right)=I-C^{T}\text{var}{\left(y_{p}\right)}^{-1}C\\ \langle v_{p}v_{p}^{T}\rangle =\text{var}\left(v_{p}|y_{p}\right)+\langle v_{p}\rangle {\langle v_{p}\rangle }^{T} \end{array}$$

In the M-step, we update *θ* and *σ*_*ε*_^2^ by maximizing Eq. (A11). To do this, we only consider the part of *F* that depends on the parameters. By taking the expectation of Eq. (A10) in respect to the distribution *q*(*v*), we get

(A13) $$\begin{array}{c} F=-\frac{N}{2}\text{log}\left|I\sigma_{\varepsilon }^{2}\right|-\frac{1}{2}\sum_{p}{\langle tr\left(v_{p}v_{p}^{T}\right)\rangle }_{q}-\frac{1}{2}tr\left({\langle S\rangle }_{q}\sigma_{\varepsilon }^{-2}\right)-...\\ {\langle S\rangle }_{q}=\sum_{p}y_{p}y_{p}^{T}-y_{p}{\langle v_{p}^{T}\rangle }_{q}C^{T}-C{\langle v_{p}\rangle }_{q}y_{p}^{T}+C{\langle v_{p}v_{p}^{T}\rangle }_{q}C^{T} \end{array}$$

From Eq. (A13) we can read off the sufficient statistics that we need to calculate from the data and the hidden parameters **V**, such that we can take derivatives of Eq. (A13) with respect to *θ*:

(A14) $$\begin{array}{l} {\langle S\rangle }_{q}=\left(S_{1}-S_{2}C^{T}-CS_{2}^{T}+CS_{3}C^{T}\right)\\ S_{1}=\sum_{n}^{}y_{p}y_{p}^{T}\\ S_{2}=\sum_{n}^{}y_{p}{\langle v_{p}^{T}\rangle }_{q}\\ S_{3}=\sum_{p}^{}{\langle v_{p}v_{p}^{T}\rangle }_{q} \end{array}$$

Given these sufficient statistics, we can now find the best solution for *θ* using linear regression. For the unconstrained case, the maximum likelihood estimators are

(A15) $$\begin{array}{c} C^{\left(u+1\right)}=\left(\sum_{p}^{}y_{p}{\langle v_{p}\rangle }^{T}\right){\left(\sum_{p}^{}\langle v_{p}v_{p}^{T}\rangle \right)}^{-1}=S_{2}S_{3}^{-1}\\ \sigma_{\varepsilon }^{2\left(u+1\right)}=\frac{1}{n}diag\left(\langle S\rangle \right) \end{array}$$

$${\langle S\rangle }_{q}=\frac{1}{N}\left(S_{1}-C^{\left(u+1\right)}S_{2}^{T}\right)$$

Because of the linear constraint (Eq. (A3)), however, we need to take the derivative of *F* (Eq. (A13)) with respect to all the parameters:

(A16) $$\begin{array}{l} \frac{\partial \langle \text{log}p_{c}\rangle }{\partial \theta_{k}}=-\frac{1}{2}tr\left[\frac{\partial {\langle S\rangle }_{q}}{\partial \theta_{k}}\right]\sigma_{\varepsilon }^{-2}\\ =-\frac{1}{2}tr\left[\left(-2C_{k}^{}S_{2}^{T}-2\sum_{j}\theta_{k}C_{k}S_{3}C_{j}^{T}\right)\right]\sigma_{\varepsilon }^{-2} \end{array}$$

By setting these derivatives to zero, we obtain the following system of linear equations:

(A17) ⋱trCkS3CjT⋱θ1⋮θK=trC1S2T⋮trCKS2T

Finally, we can simply invert this equation to obtain the maximum likelihood estimate of the parameters given the sufficient statistics of **V**. By iterating the E and M steps, the expected log-likelihood is guaranteed to increase with every step, thereby increasing the lower bound on the likelihood.

### Implementation

The algorithm is implemented in the Matlab function mvpattern_covcomp.m, which can be found at “http://www.icn.ucl.ac.uk/motorcontrol/imaging/multivariate_analysis.htm” together with example code that reproduces all simulations reported in this paper.

To speed the convergence, the algorithm uses the Aitken acceleration method (McLachlan and Krishnan, 1997). After 3 normal EM iterations, the scheme looks at the first and second derivative of the convergence and tries a large jump to the predicted best value. Because an increase in likelihood is not assured after such jumps, the increase needs to be tested and the jump rejected if the likelihood decreased. The method speeds convergence substantially. For the example of the one-factorial design (2.2) convergences was reached on average after 28 iterations or 15 ms (Quad-core Apple Pro, 2.26GHz), on the example of the two-factorial design (3.1), convergence was achieved after 86 iterations and 90 ms. For the latter example, the scheme is about a factor of 3 faster than a standard Fisher-scoring scheme (Friston, 2008).

## Footnotes

1 While the approach presented here assumes temporally independent error terms, an extension to times-series analysis with auto-correlated errors is possible by incorporating a structured covariance matrix in Eqs. (A16) and (A17).
